# A Century of Tuberculosis Epidemiology in the Northern and Southern Hemisphere: The Differential Impact of Control Interventions

**DOI:** 10.1371/journal.pone.0135179

**Published:** 2015-08-19

**Authors:** Sabine Hermans, C. Robert Horsburgh Jr., Robin Wood

**Affiliations:** 1 Desmond Tutu HIV Centre, Institute of Infectious Disease and Molecular Medicine, University of Cape Town, Cape Town, South Africa; 2 Department of Global Health, Academic Medical Center, University of Amsterdam, Amsterdam Institute for Global Health and Development, Amsterdam, the Netherlands; 3 Department of Internal Medicine, School of Medicine, Makerere University College of Health Sciences, Kampala, Uganda; 4 Department of Epidemiology, Boston University School of Public Health, Boston, Massachusetts, United States of America; 5 Department of Medicine, University of Cape Town, Cape Town, South Africa; 6 Department of Clinical Research, Faculty of Infectious & Tropical Diseases, London School of Hygiene & Tropical Medicine, London, United Kingdom; University of Cape Town, SOUTH AFRICA

## Abstract

**Background:**

Cape Town has one of the highest TB burdens of any city in the world. In 1900 the City of Cape Town, New York City and London had high mortality of tuberculosis (TB). Throughout the 20th century contemporaneous public health measures including screening, diagnosis and treatment were implemented in all three settings. Mandatory notification of TB and vital status enabled comparison of disease burden trajectories.

**Methods:**

TB mortality, notification and case fatality rates were calculated from 1912 to 2012 using annual TB notifications, TB death certifications and population estimates. Notification rates were stratified by age and in Cape Town by HIV status (from 2009 onwards).

**Results:**

Pre-chemotherapy, TB mortality and notification rates declined steadily in New York and London but remained high in Cape Town. Following introduction of combination chemotherapy, mean annual case fatality dropped from 45–60% to below 10% in all three settings. Mortality and notification rates subsequently declined, although Cape Town notifications did not decline as far as those in New York or London and returned to pre-chemotherapy levels by 1980. The proportional contribution of childhood TB diminished in New York and London but remained high in Cape Town. The advent of the Cape Town HIV-epidemic in the 1990s was associated with a further two-fold increase in incidence. In 2012, notification rates among HIV-negatives remained at pre-chemotherapy levels.

**Conclusions:**

TB control was achieved in New York and London but failed in Cape Town. The TB disease burden trajectories started diverging before the availability of combination chemotherapy in 1952 and further diverged following the HIV epidemic in 1990. Chemotherapy impacted case fatality but not transmission, evidenced by on-going high childhood TB rates. Currently endemic TB results from high on-going transmission, which has been exacerbated by the HIV epidemic. TB control will require reducing transmission, which is inexorably linked to prevailing socio-economic factors.

## Introduction

TB was the largest single cause of death in industrialized countries in the mid-nineteenth century [1, 2]. TB mortality declined dramatically subsequently, preceding the identification of *Mycobacterium tuberculosis*, the sanatorium movement and the introduction of chemotherapy. This decline has been variously attributed to improving social circumstances, segregation of infectious cases in sanatoria and to natural selection [1, 3–5]. Following introduction of chemotherapy in the 1950’s, TB mortality and notification rates continued to improve.

In less developed parts of the world however, TB remains a major cause of morbidity and mortality. South Africa reports the highest TB notification rate in the world, and TB is its leading cause of natural death [6, 7].

For two centuries the epidemiology of tuberculosis (TB) has been largely viewed through the experiences of industrialized countries of the northern hemisphere [1]. The introduction of vital status registration in the nineteenth century and compulsory TB case notification in the early twentieth century enabled tracking of national disease burdens. Historical data from currently high-burdened countries are scarce; India, for example, only made TB a notifiable disease in 2012 [8]. Cape Town, facing a high TB burden at the beginning of the 20^th^ century, became one of the first cities to introduce compulsory notification in 1904 [9].

A century of TB case and death registrations in Cape Town, New York and London offers a unique opportunity to compare secular changes resulting in differing levels of TB control. Over the last century the City of Cape Town (Cape Town) has remained a single administrative unit, reporting all cases and deaths within its jurisdiction. Records of the Medical Officers of Health (MoH) from 1912 to 2012 in Cape Town, New York City (New York) and London were reviewed to explore trends in TB burden and temporal associations with TB control strategies, the introduction of chemotherapy and the advent of the HIV epidemic.

## Methods

### Population

Estimated mid-year populations of Cape Town were extracted from the MoH annual reports and the National Department of Health Information System Programme [9, 10]. HIV prevalence was estimated from the Actuarial Society of South Africa Western Cape AIDS and Demographic model 2008 [11]. The annual mid-year populations of New York 1912–1950 were sourced from the report of the New York Tuberculosis and Health Organisation [2], thereafter calculated from the 10-yearly censuses of New York [12, 13]. Cape Town and New York’s administrative definition remained the same over time. In 1965, London County Council was combined with Outer London to form the Greater London Area. As this became the administrative unit used for censuses and reporting of notifiable diseases, we therefore defined London as London County Council up to 1965 and as the Greater London Area from then onwards. The mid-year population estimates for London using these definitions were calculated using data from the 11 censuses conducted during this period [14, 15].

### TB mortality

Annual TB death certifications were abstracted from the MoH annual reports (Cape Town) and the annual summaries of the Bureau of Tuberculosis Control (New York) [9, 16]. London TB death notifications were sourced from the London MoH annual reports for London County Council (1913–1964), the Registrar-General's Annual Statistical Review (1965–1973), the Office for Population Censuses and Surveys (OPCS) Monitor Mortality Statistics (1974–1992) and the Compendium of Population Health Indicators (1992–2012) [17–20].

### TB case notifications

Cape Town TB case notifications were obtained from the MoH annual reports, the city TB programme progress report and the electronic TB register of the TB control programme [9, 21]. TB notifications were available by population group until 1994 by age from 1930 onwards (except 1994–2001). TB notifications were stratified by HIV status for 2009–2012, when over 90% of TB cases were HIV tested. New York TB case notifications were sourced from annual summaries of the Bureau of Tuberculosis Control [16]. London TB notification numbers were abstracted from the London MoH annual reports (1913–1964), the Registrar-General's Annual Statistical Review (1965–1973), the Office for Population Censuses and Surveys (OPCS) Monitor Communicable Diseases Statistics (1974–1981) and from Public Health England (1982–2012) [17, 18, 22–24]. London TB notifications 1965–1973 were published in rates, which were calculated back to numbers of cases using population data. All TB notifications were included irrespective of their method of diagnosis, which was not consistently reported. Non-pulmonary TB notifications were not available for New York 1912–1920 and London from 1965 onwards.

### TB control programme interventions

The timing of public health interventions was determined by examining the annual reports of the Cape Town and London MoH and the New York Bureau of Tuberculosis Control, complemented by other sources including books, electronic databases, dissertations and theses [2, 9, 16, 25–33].

### Statistical methods

Population denominators were interpolated for intervening years between censuses [34]. All data are included in the supporting information (S1 Dataset). We calculated TB notification and mortality rates per 100,000 population by 5-year period from 1912 to 2012. Age-stratified TB notification rates were calculated for the years available. The Cape Town TB notification rates were stratified by HIV infection for the years that the HIV status of >90% of TB cases was reported (2009–2012). We estimated the yearly TB case fatality rate by dividing the annual deaths by the annual TB notifications [35]. Differences in rate changes over time were calculated using linear regression. All analyses were conducted using Microsoft Excel 2010 (Redmond, Washington, USA) and STATA 13.0 IC (College Station, Texas).

### Ethics statement

This analysis was performed using publicly available data, which is considered exempt from research ethics review according to the Standard Operating Procedures of the University of Cape Town Human Research Ethics Committee.

## Results

Between 1912 and 2012, Cape Town’s population increased from 79 thousand to 3.6 million due to geographic expansion and immigration from Europe and the surrounding rural areas. Over the same period, the population of New York grew from 4.9 million to 8.2 million and London from 5.0 million to 8.3 million. In 1912 the population age-pyramids of the three settings were similar (S1 Fig). By 2012, the populations of New York and London had aged due to increased life expectancy and a decreased birth rate. Cape Town’s population had also aged, although to a lesser extent. Cape Town’s growth stratified by population group reflects its geopolitical history (S2 Fig). The European and Coloured population grew at a similar rate until the 1950’s. Thereafter the growth was mainly among the Coloured population, due to lower mortality, increased birth rates and immigration from rural areas in the Western Cape province [36]. After the end of apartheid in 1994 various adjacent municipalities were incorporated into the city. Africans were a minority until that time, when Cape Town saw a surge in migrants from the Eastern Cape province of South Africa.

TB control measures were implemented contemporaneously in Cape Town and London, which had been the colonial power, and New York (Table 1). Vital status registration, mandatory TB notification and sputum smear microscopy for diagnosis of TB suspects were conducted in all three settings. In Cape Town, in response to the very high burden of TB among infants, BCG vaccination was implemented in neonates. In New York BCG was not implemented as a generalised public health measure.

**Table 1:** Timeline of implementation of TB control interventions in Cape Town, New York and London. Data from [2, 9, 16, 25–33].

Intervention	Cape Town	New York	London
Birth & death registration	1894	1804	1836
Notification	1904	1897	1912
Dispensary triage	1910	1904	1909
Tuberculin therapy	1912	N/A	1914
Artificial pneumothorax	1924	1930	1920
First sanatorium opened	1924	1903	1899
Tuberculin testing of cattle	1908	1919	1911
Milk pasteurisation	1930s (V), 1953 (M)	1912 (M)	1930s (V)
Mass radiography	1948	1932	1943
Streptomycin/PAS/Isoniazid	1953	1953	1952
BCG vaccination	1959 (neonatal)	N/A	1953 (school children)
Short course rifampicin therapy	1980	1981	1976
Public sector ART	2001	1996	1996

ART, antiretroviral therapy; BCG, Bacille Calmette-Guérin; M, mandatory; N/A, not applicable; PAS, P-aminosalicylic acid; V, voluntary.

**Fig 1:**
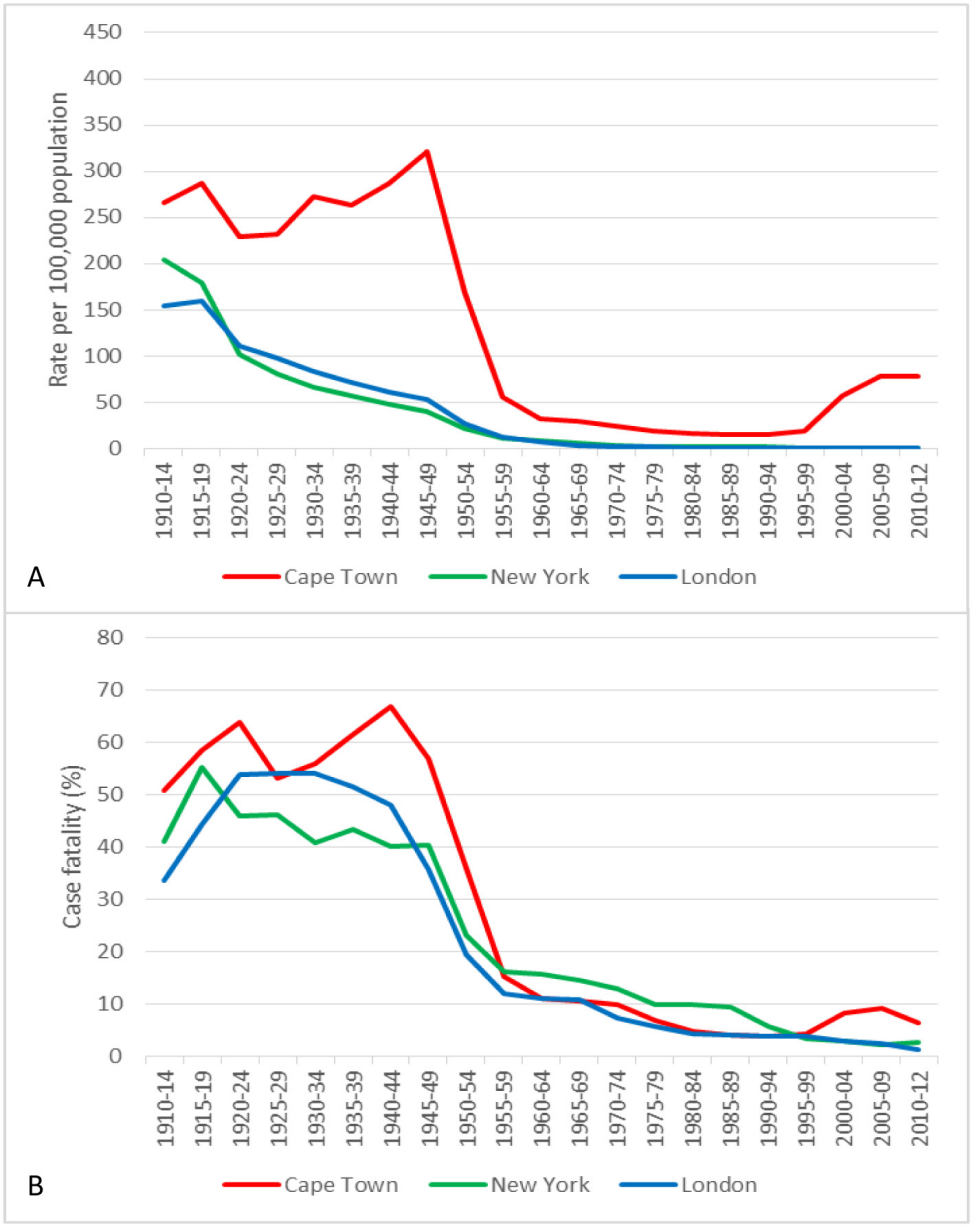
TB mortality and case fatality rates over time. A. TB mortality rates over time. B. TB case fatality rates over time. Note. Rates from 1913 to 1965 are for London County Council (current Inner London) [17], thereafter for the Greater London Area (Inner and Outer London)[23, 24]. CT, Cape Town; NY, New York.

TB mortality rates in Cape Town remained at high levels during the pre-chemotherapy era (250–300 per 100,000, Fig 1A), similar to those recorded in London in the second half of the 19^th^ century [3]. By 1913, TB death rates in London had already decreased to 100 per 100,000 and continued to decline over the next century. Mortality rates in New York showed a similar pattern delayed by 20 years [2]. Cape Town saw mortality decrease by 80% between 1950 and 1960. During the same period the case fatality rate plummeted almost simultaneously in all three settings (Fig 1B).

**Fig 2:**
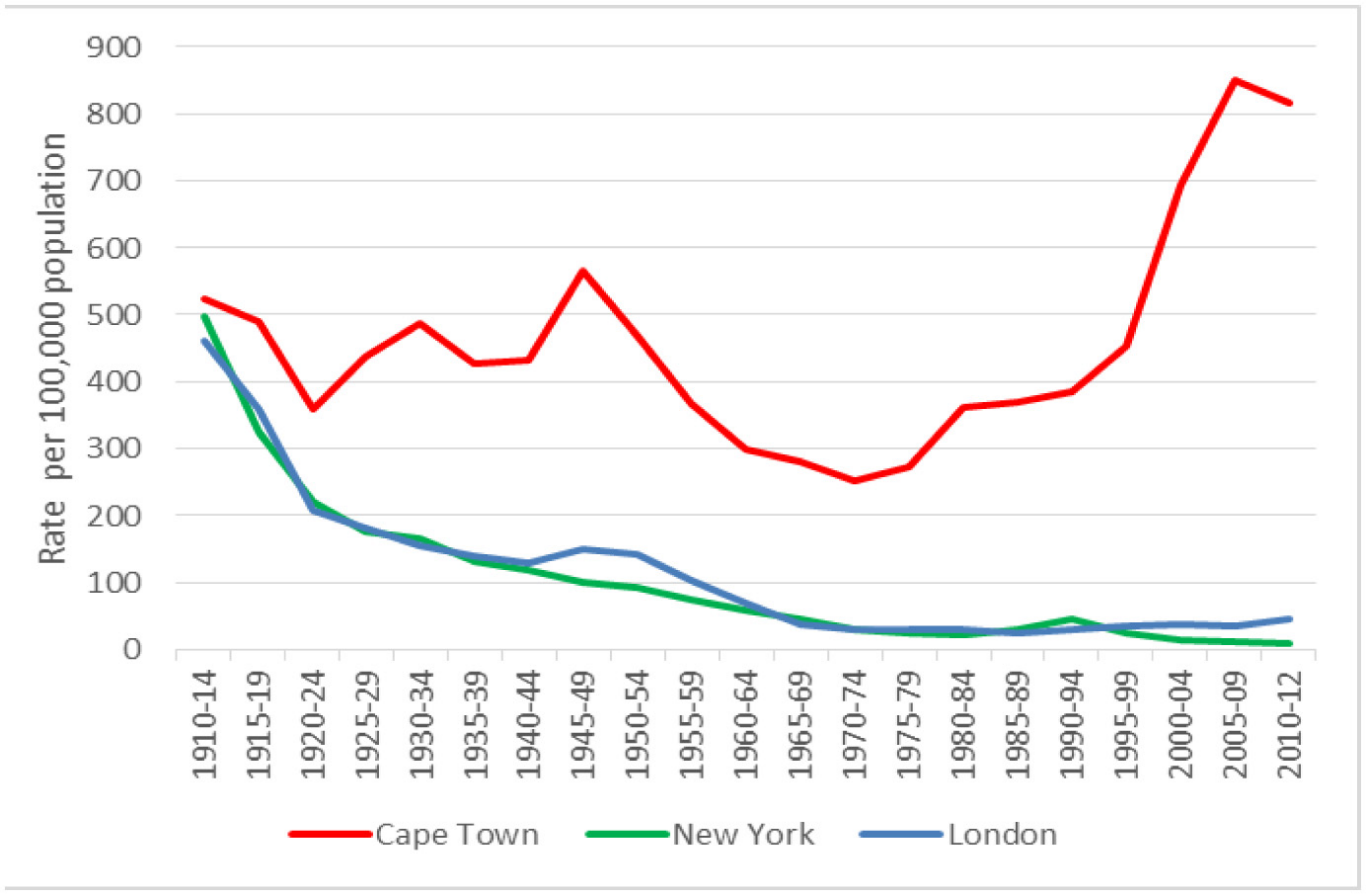
TB notification rates over time. The current (2009–2012) HIV-negative rate in Cape Town was 445 per 100,000 population, the HIV-positive rate was 6338 per 100,000 population (not shown). Note. Rates from 1913 to 1965 are for London County Council (current Inner London) [17], thereafter for the Greater London Area (Inner and Outer London) [23, 24]. CT, Cape Town; NY, New York.

The Cape Town TB notifications fluctuated around 450 cases per 100,000 population between 1910 and 1945 (Fig 2). After the introduction of chemotherapy they decreased to a nadir of 250 cases per 100,000 in 1970, increased again to 450/100,000 in 1995 and doubled with the HIV epidemic to 850 per 100,000 in 2010. The HIV-negative TB notification rate 2009–2012 was 445 per 100,000, versus 6338 per 100,000 among the HIV-positives. In contrast, TB notification rates in London and New York showed a steady decline throughout the century. The Second World War saw a transient increase in TB notifications in London and to a lesser extent New York, after which rates declined further.

**Fig 3:**
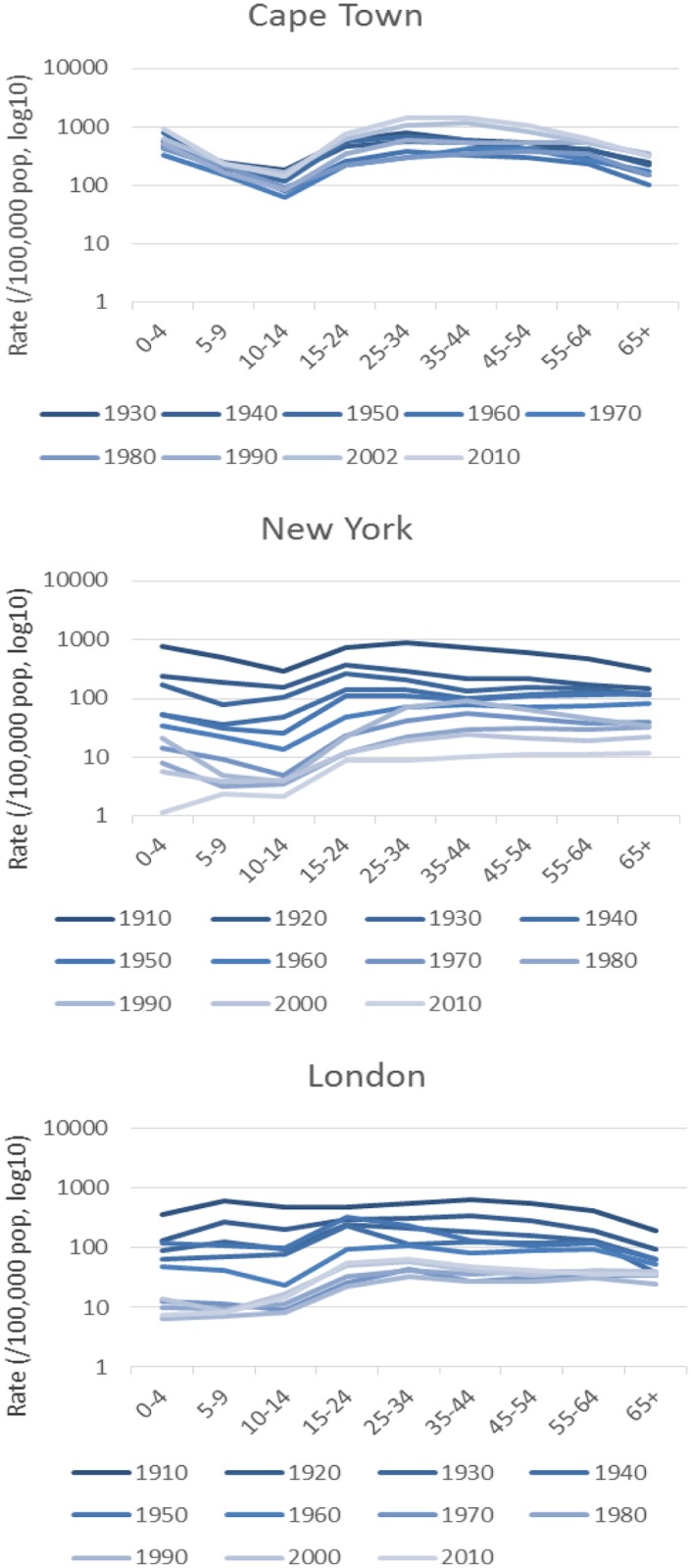
Age-stratified TB notification rates per decade over time. Note. Y-axes are on a logarithmic scale (base 10). Age-stratified rates in Cape Town were not available prior to 1930. Cape Town rates for 2002 and 2010 include TB in HIV-infected persons.

**Fig 4:**
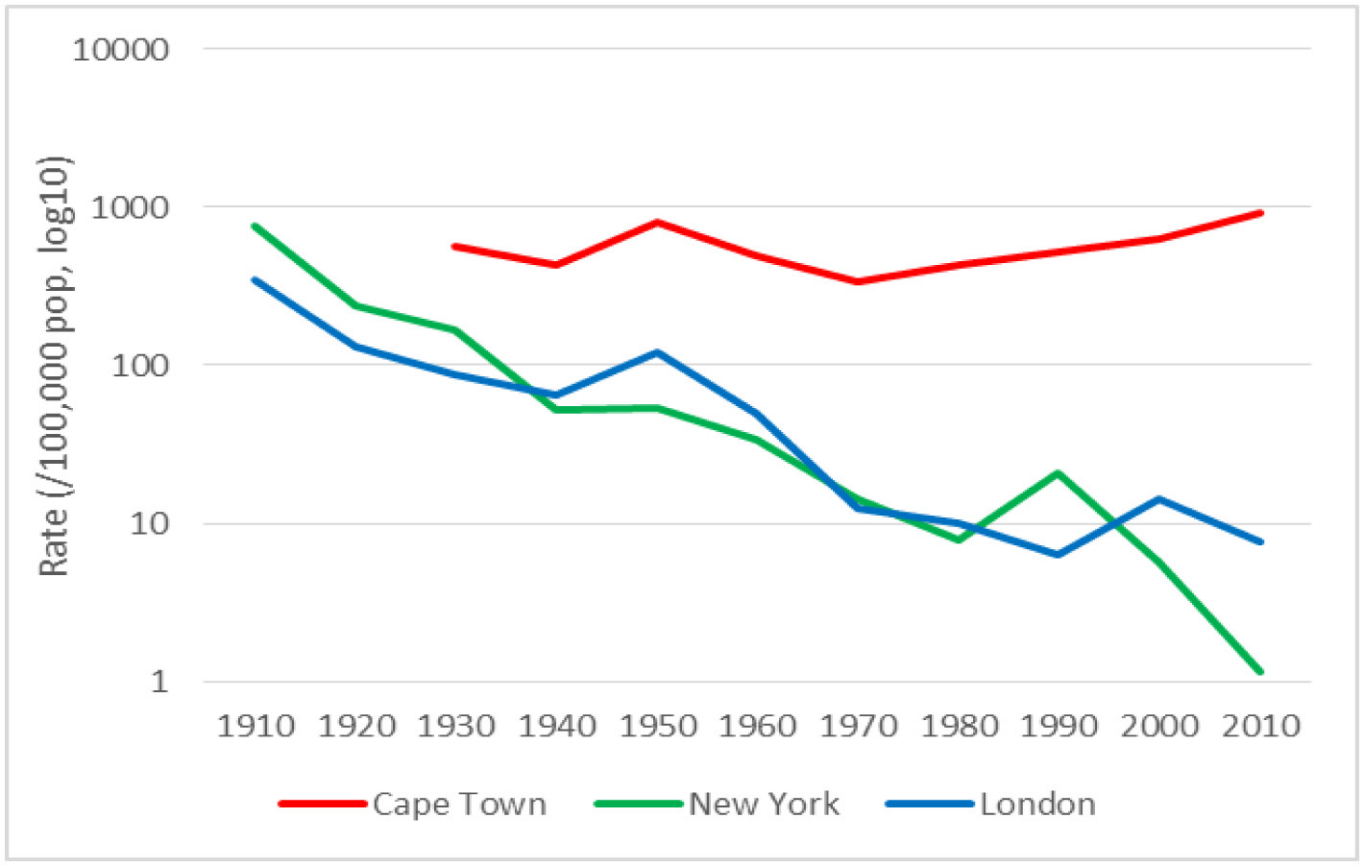
TB notification rates among children aged 0–4 years per decade over time. Note. Y-axes are on a logarithmic scale (base 10). Age-stratified rates in Cape Town were not available prior to 1930. Cape Town rates for 2002 and 2010 include TB in HIV-infected persons.

In Cape Town, the distribution of TB notifications among the different age strata remained stable over time (Fig 3). In New York and London, however, the proportional contribution of childhood TB to the overall disease burden greatly reduced over time (Fig 4, S1 Table and S3 Fig). Cape Town TB notification rates in the under-fives are currently at the same level as or higher than at the beginning of the century. There was good evidence for a difference in rate change over time between Cape Town on the one hand and New York and London on the other (*P* = 0.045 and *P* = 0.026), and no evidence for a difference between New York and London (*P* = 0.81).

Cape Town’s TB mortality and notification rates stratified by population group showed similar trends in case fatality (S4 Fig). However, the temporary decline in TB notification rates was mainly seen in the Non-European population.

In the 1980’s, a low prevalence HIV epidemic was present in all three settings [37]. In Cape Town in the early 1990’s there was a rapid growth of a heterosexually transmitted epidemic [38]. In 1994 antenatal HIV prevalence reached 1%, after which it increased to 18% in 2010 and has subsequently remained stable [39]. Following the onset of the HIV epidemic TB mortality reversed and increased 5-fold (from 15 to 79 per 100,000). Both New York and London reported increases in TB notification rates probably related to HIV in the 1980’s, albeit still very low in comparison to the first part of the century and to Cape Town. In addition to HIV, declining TB control programmes, poor social conditions and immigration from high-burden countries were thought to have played an important role [24, 40–42]. Increased TB control efforts led to a rapid decrease in rates in New York. London’s rates have not yet returned to their earlier levels.[43]

## Discussion

Long-term historical data offered the unique opportunity to compare TB disease burden trajectories between three settings with a high TB mortality at the start of the 20th century. Over the next century very different trajectories were recorded in Cape Town compared to New York and London. This comparison allows for important insights into TB epidemiology which may have implications for TB control in currently high burdened settings.

In all three cities, there appeared to be a temporal relationship between the introduction of combination chemotherapy and decreases in TB mortality and case fatality. The rapidity and magnitude of the decline indicates that chemotherapy was rapidly provided to the majority of TB cases in all three settings. In Cape Town there was a clear decrease in TB mortality in both the European and non-European populations between 1950 and 1954, coincident with the introduction of combination chemotherapy. A similar temporal trend was seen in New York. However, in London some decrease in mortality occurred between 1945 and 1949 which may have been attributable to non-treatment factors in the period immediately following the turmoil of the Second World War.

Historically, mortality rates have always been used as a measure of TB burden. However, the impact of chemotherapy on TB notification rates was much less clear than on mortality. There were ongoing declines in notifications in New York and London but there were persistent high rates in Cape Town after an initial period of decline.

TB control strategies were similar and contemporaneous in the three cities. In addition New York employed contact tracing and immigrant screening with isoniazid treatment of those found to be infected from the 1970’s onwards [16]; these measures were used less in London [43]. Since London and NYC had similar trajectories over the century, these differences are not likely to explain the marked differences between Cape Town and the two northern cities.

An important difference between Cape Town and the other two cities was the trajectory of TB notifications in children. While paediatric TB all but disappeared in New York and London, it remained unchanged in Cape Town. The introduction of chemotherapy had almost no impact on childhood TB in Cape Town. As TB notifications among young children are a result of recent acquisition of TB infection, this is a strong indication that TB transmission remained high throughout. This results in a majority of children acquiring TB infection before adulthood [44]; in contrast, in London the majority reach adulthood without becoming infected [45].

Mortality is a function of case fatality and TB incidence. As prevailing medical interventions before the advent of chemotherapy did not majorly impact case fatality, the declining mortality rates in New York and London likely reflected decreasing incidence. In contrast, the high mortality rates in Cape Town reflected on-going high incidence which has continued to this day. Despite the doubling of the Cape Town TB burden after the advent of HIV, HIV-negative TB notification rates remained stable at pre HIV-epidemic levels. Therefore the TB epidemic was already poorly controlled at that time, challenging the widespread belief that the HIV epidemic is solely responsible for the current high TB rates. Rather than being the cause of transmission itself, it exposed a pre-existing high transmission rate.

There are several factors that may have contributed to the upward trend in Cape Town notification rates from the 1970’s onwards. The temporary decrease in TB notification rates between 1950 and 1970 coincided with a period of mass active case finding using miniature radiography, during which the working European and non-European populations were intensively screened for TB. During that period more than 10% of the total population was screened by miniature mass radiography annually [9].

Population growth was strikingly different between the three cities with Cape Town growing much more rapidly in this period. Additionally the proportion of the heavily burdened non-European population also increased in Cape Town, predominantly due to an increase in the mixed-race population that originate from the areas immediately around Cape Town. In contrast, black immigration increased rapidly after 1994 as a result of freedom of movement with the new political dispensation.

Increased survival due to effective treatment in the 1950’s may also have contributed to increasing TB notification rates. The numbers of individuals with prior successfully treated TB would have been expected to progressively increase over time. These TB survivors, now at risk of re-infection, would increasingly contribute to the overall TB burden over time. Recurrent TB disease, unrecognised before the 1980’s, currently constitutes 26% of Cape Town TB notifications of which the majority have no indication of default from initial therapy [46].

Underlying socioeconomic, biological and environmental conditions could make the population of Cape Town more at risk of TB than that of New York and London. Cape Town has high inequality with the most recent equity index estimated at 0.22 compared to 0.50 in New York and 0.79 in London [47]. Lower socioeconomic circumstances are associated with crowding and malnutrition, known risk factors for TB transmission and progression to active disease. The effective contact number in Cape Town might have remained high, where it declined dramatically in London over the last century [48]. During that time average household sizes in the United States and Britain decreased 2-fold [49, 50]. Historical data on average household size in Cape Town are not available, but it is currently higher in Cape Town than in New York and London (3.5 versus 2.4 and 2.5) [50–52]. The increased in-migration may have caused more crowded conditions, with increased spread of TB as a consequence. A higher genetic susceptibility for TB infection and disease is unlikely considering that TB has affected all population groups in Cape Town at different times throughout the century. Finally, there could be environmental differences between Cape Town and the northern cities which facilitate TB transmission.

Our results demonstrate that Cape Town has remained a high-burden setting despite the availability of all contemporary TB control measures, including chemotherapy. These measures were implemented when TB incidence rates were already declining in New York and London; simultaneous implementation in Cape Town where rates were still high had less impact. Chemotherapy only affected case fatality with little impact on TB incidence. Other current high-burden countries may have had similar epidemiologic trajectories, but lack data to demonstrate this. There is a clear need to better understand how and where TB is being transmitted, and which interventions, whether social, such as ensuring separate sleeping spaces for children, environmental, such as maintenance of adequate ventilation in transport and schools, or medical, such as screening of young adults and household contacts, are best suited to interrupt transmission. Current TB control programmes need to be augmented with such strategies in order for the epidemic to be curbed.

Strengths of our analysis include the length of the period studied (100 years). The similarity in the TB control strategies implemented and the timing thereof is striking and justifies the comparison we present. Also, the comparison of secular trends between three settings with a high TB burden at the start of the 20th century, two of which successfully brought the disease under control whereas the other still suffers from an extremely high burden, has not been made previously.

Our results should be interpreted in the light of limitations. Rates were not age or sex standardised because stratified data were not consistently available. The population age-structure remained relatively stable in Cape Town, minimising the impact of this on our findings. Non-pulmonary TB case notifications were not available for New York 1912–1920 and London from 1965 onwards. However, the proportional contribution of extra-pulmonary TB in London decreased from 25% in 1913 to 11% in the 1965, and was minimal in New York during the second half of the century. Lastly, data collection over such long periods of time is subject to differing case definitions, reporting and ascertainment biases, which we could not evaluate due to lack of data. Throughout the century, Cape Town has had good laboratory services offering TB cultures as routine part of TB diagnosis as soon as they became available. We did not have data on case detection for the period studied. If this were consistently lower in Cape Town than in New York or London, this might have resulted in an excess of untreated cases in Cape Town compared to the other cities.

## Conclusions

TB control was achieved in New York and London but failed in Cape Town despite the implementation of contemporaneous measures. The diverging disease burden trajectories starting in the pre-chemotherapy era suggest social and environmental differences may be associated with the propagation of endemic TB in Cape Town. Combination chemotherapy dramatically improved case fatality with little effect on on-going transmission. More recently the generalised HIV epidemic has further exposed the failure to control TB transmission. A better understanding of TB transmission and how to interrupt it is key to curbing endemic TB, and such strategies will need to be incorporated into TB control programmes of high-burden settings.

## Supporting Information

S1 Dataset Annual TB notifications, TB deaths and mid-year population for Cape Town, New York and London, 1912–2012. (XLSX)

S1 Fig Population pyramids of Cape Town, New York and London in 1912 and 2012. Note. The X-axes represent proportions of the population. Data from [2, 9, 10, 12–15].
(TIF)

S2 Fig Cape Town population over time stratified by population group. (TIF)

S3 Fig TB notification rates by 5 year age group per decade over time in Cape Town, New York and London. (TIF)

S4 Fig Cape Town TB notification rate (A) and TB mortality rate (B) over time stratified by population group. (TIF)

S1 Table Age-stratified TB notification rates per decade over time in Cape Town, New York and London. Note. Age-stratified rates in Cape Town were not available prior to 1930. Cape Town rates for 2002 and 2010 include TB in HIV-infected persons.
(DOCX)
