# Supplementary figures and images for: A Century of Tuberculosis Epidemiology in the Northern and Southern Hemisphere: The Differential Impact of Control Interventions
Source: PLoS One. 2015 Aug 19;10(8):e0135179. doi: 10.1371/journal.pone.0135179 (PMC4545605; doi:10.1371/journal.pone.0135179)

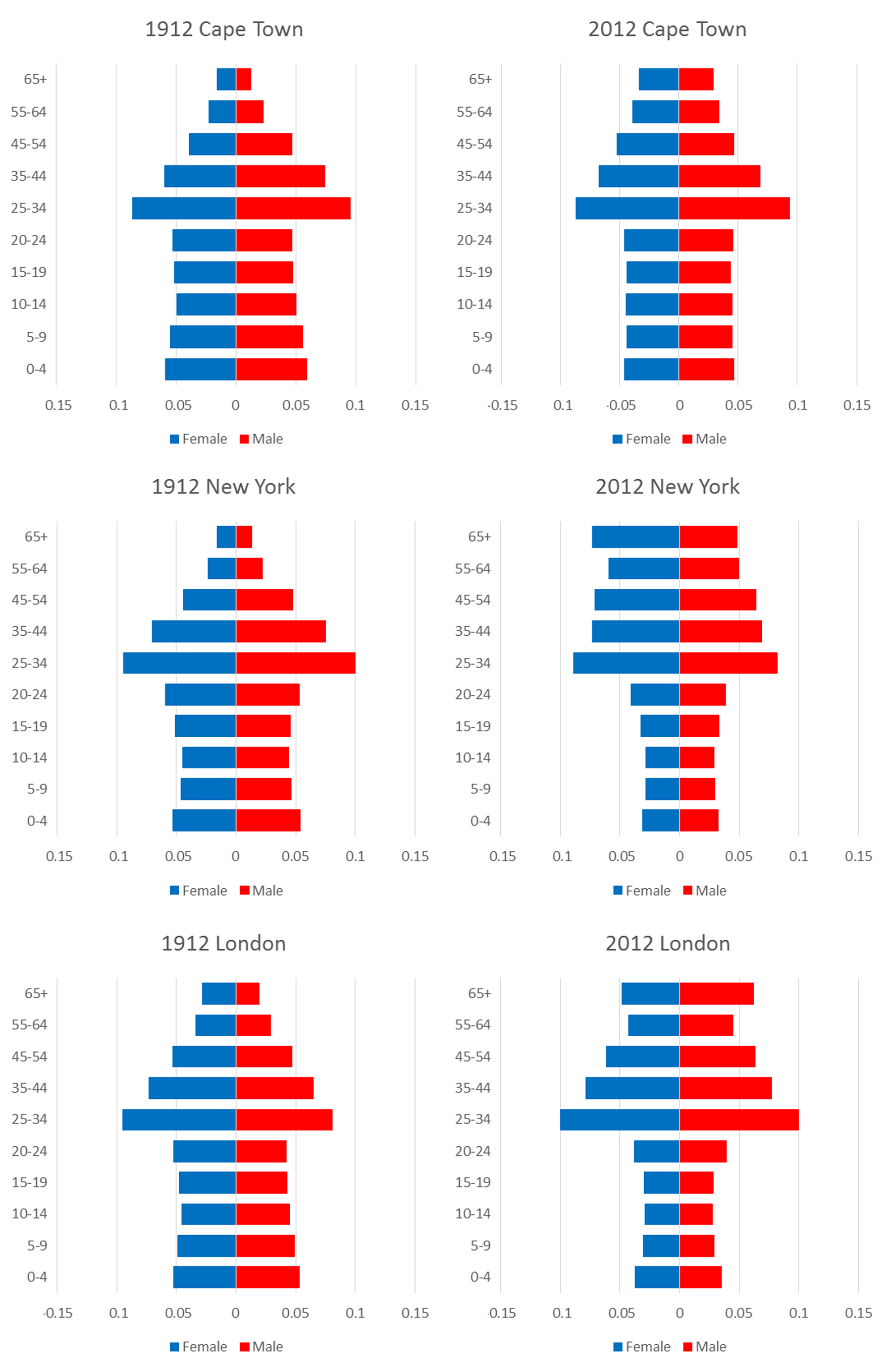

Supplement: S1 Fig — Note. The X-axes represent proportions of the population. Data from [2, 9, 10, 12–15]. (TIF) [file pone.0135179.s002.tif]

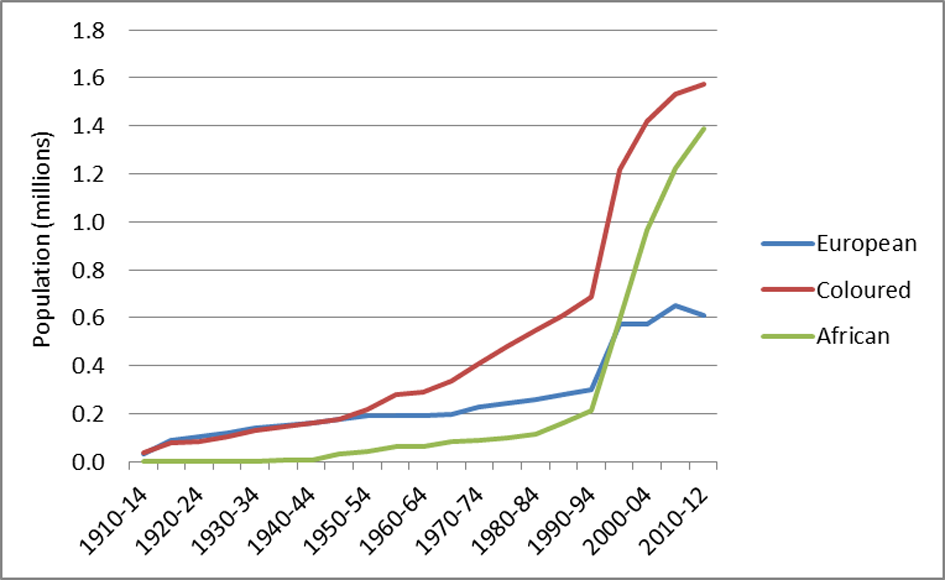

Supplement: S2 Fig — (TIF) [file pone.0135179.s003.tif]

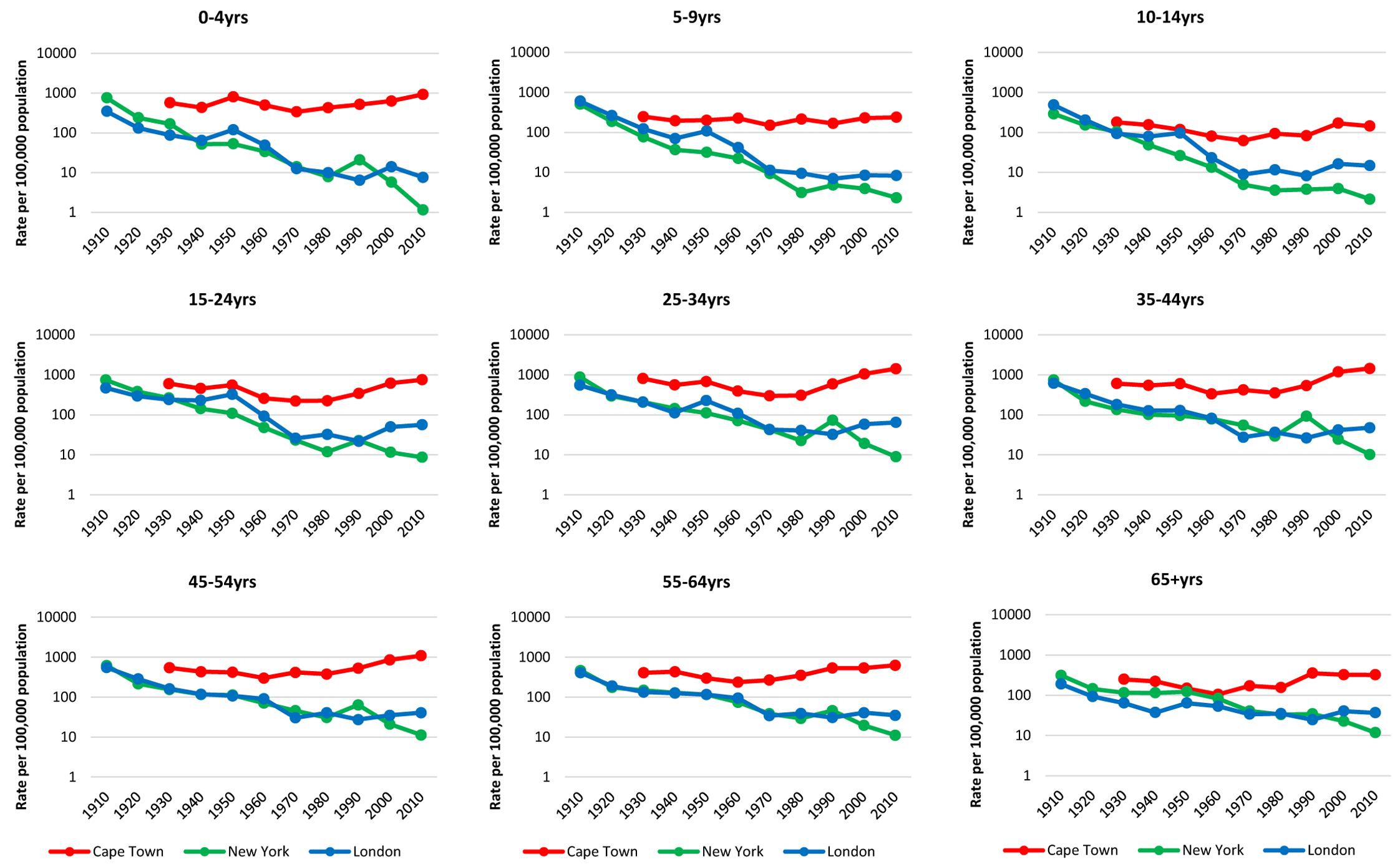

Supplement: S3 Fig — (TIF) [file pone.0135179.s004.tif]

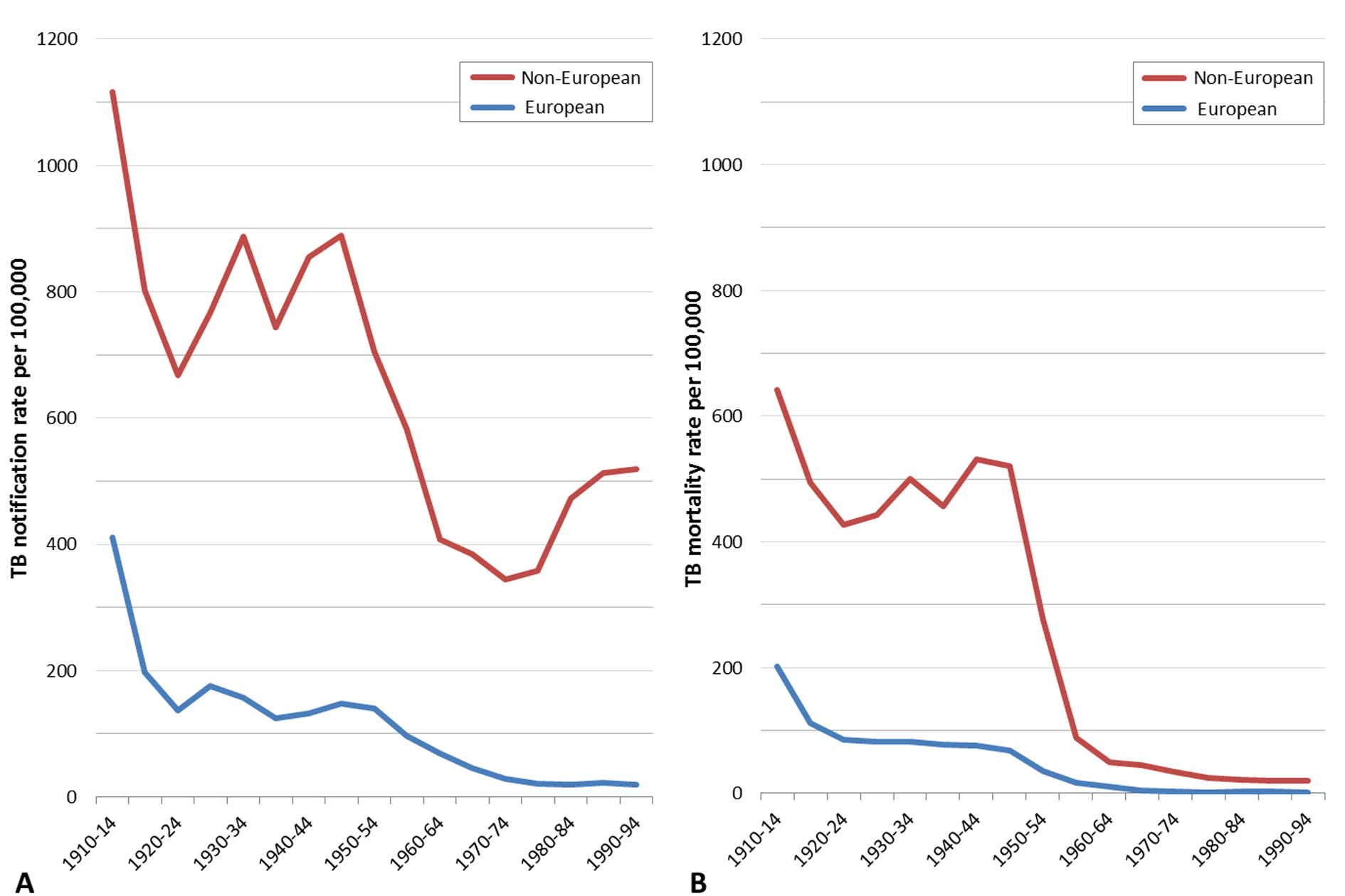

Supplement: S4 Fig — (TIF) [file pone.0135179.s005.tif]
