# Supplementary material for: A Century of Tuberculosis Epidemiology in the Northern and Southern Hemisphere: The Differential Impact of Control Interventions
Source: PLoS One. 2015 Aug 19;10(8):e0135179. doi: 10.1371/journal.pone.0135179 (PMC4545605; doi:10.1371/journal.pone.0135179)
Supplement: S1 Table — Note. Age-stratified rates in Cape Town were not available prior to 1930. Cape Town rates for 2002 and 2010 include TB in HIV-infected persons. (DOCX) [file pone.0135179.s006.docx]

**Table S1. Age-stratified TB notification rates per decade over time in Cape Town, New York and London.**

| **City** | **Year** | **Rates (/100,000 population) per 5 year age category** | | | | | | | | |
| --- | --- | --- | --- | --- | --- | --- | --- | --- | --- | --- |
|  |  | **0-4** | **5-9** | **10-14** | **15-24** | **25-34** | **35-44** | **45-54** | **55-64** | **65+** |
| Cape Town | 1930 | 572 | 248 | 180 | 603 | 804 | 607 | 541 | 406 | 248 |
|  | 1940 | 433 | 197 | 154 | 455 | 557 | 541 | 431 | 432 | 221 |
|  | 1950 | 808 | 202 | 117 | 555 | 679 | 603 | 419 | 297 | 148 |
|  | 1960 | 498 | 226 | 80 | 257 | 392 | 333 | 299 | 236 | 105 |
|  | 1970 | 339 | 150 | 62 | 222 | 297 | 418 | 414 | 267 | 171 |
|  | 1980 | 432 | 216 | 93 | 225 | 306 | 352 | 378 | 350 | 154 |
|  | 1990 | 520 | 167 | 83 | 344 | 590 | 538 | 529 | 536 | 353 |
|  | 2002 | 635 | 230 | 170 | 619 | 1050 | 1181 | 858 | 533 | 324 |
|  | 2010 | 930 | 239 | 144 | 755 | 1422 | 1440 | 1087 | 623 | 321 |
| New York | 1910 | 761 | 508 | 291 | 745 | 881 | 744 | 617 | 463 | 310 |
|  | 1920 | 240 | 189 | 153 | 379 | 294 | 218 | 215 | 174 | 144 |
|  | 1930 | 168 | 77 | 104 | 262 | 208 | 136 | 155 | 148 | 116 |
|  | 1940 | 52 | 37 | 49 | 143 | 143 | 101 | 116 | 128 | 115 |
|  | 1950 | 53 | 32 | 26 | 109 | 111 | 96 | 112 | 116 | 122 |
|  | 1960 | 34 | 22 | 13 | 48 | 72 | 78 | 71 | 74 | 82 |
|  | 1970 | 14 | 9 | 5 | 23 | 43 | 55 | 46 | 38 | 41 |
|  | 1980 | 8 | 3 | 4 | 12 | 23 | 29 | 31 | 29 | 33 |
|  | 1990 | 21 | 5 | 4 | 23 | 73 | 92 | 64 | 46 | 34 |
|  | 2000 | 6 | 4 | 4 | 12 | 19 | 24 | 21 | 20 | 23 |
|  | 2010 | 1 | 2 | 2 | 9 | 9 | 10 | 11 | 11 | 12 |
| London | 1910 | 349 | 607 | 485 | 470 | 549 | 618 | 548 | 408 | 189 |
|  | 1920 | 132 | 266 | 204 | 293 | 315 | 336 | 286 | 189 | 93 |
|  | 1930 | 88 | 122 | 93 | 239 | 207 | 181 | 161 | 134 | 64 |
|  | 1940 | 65 | 71 | 79 | 229 | 112 | 127 | 118 | 126 | 37 |
|  | 1950 | 120 | 108 | 96 | 325 | 227 | 129 | 107 | 116 | 64 |
|  | 1960 | 49 | 42 | 23 | 93 | 109 | 81 | 91 | 94 | 53 |
|  | 1970 | 13 | 11 | 9 | 26 | 43 | 28 | 30 | 34 | 34 |
|  | 1980 | 10 | 9 | 12 | 32 | 41 | 36 | 40 | 39 | 35 |
|  | 1990 | 6 | 7 | 8 | 22 | 32 | 26 | 27 | 31 | 24 |
|  | 2000 | 14 | 9 | 16 | 50 | 58 | 42 | 35 | 41 | 40 |
|  | 2010 | 8 | 8 | 15 | 56 | 64 | 48 | 41 | 35 | 37 |

Note. Age-stratified rates in Cape Town were not available prior to 1930. Cape Town rates for 2002 and 2010 include TB in HIV-infected persons.
